# *Lophelia pertusa* corals from the Ionian and Barents seas share identical nuclear ITS2 and near-identical mitochondrial genome sequences

**DOI:** 10.1186/1756-0500-6-144

**Published:** 2013-04-11

**Authors:** Jean-François Flot, Mikael Dahl, Carl André

**Affiliations:** 1 Max Planck Institute for Dynamics and Self-Organization, Biological Physics and Evolutionary Dynamics, Göttingen, 37077, Germany; 2 Department of Biological and Environmental Sciences – Tjärnö, University of Gothenburg, Strömstad, 452 96, Sweden

**Keywords:** Mitogenomics, Control region, Internal transcribed spacer, Haploweb, Phylogeography, Mediterranean outflow water

## Abstract

**Background:**

*Lophelia pertusa* is a keystone cold-water coral species with a widespread distribution. Due to the lack of a mitochondrial marker variable enough for intraspecific analyses, the population structure of this species has only been studied using ITS and microsatellites so far. We therefore decided to sequence and compare complete mitochondrial genomes from two distant *L. pertusa* populations putatively isolated from each other (in the Barents Sea off Norway and in the Mediterranean Sea off Italy) in the hope of finding regions variable enough for population genetic and phylogeographic studies.

**Results:**

The mitogenomes of two *L. pertusa* individuals collected in the Mediterranean and Barents seas differed at only one position, which was a non-synonymous substitution, but comparison with another recently published *L. pertusa* mitochondrial genome sequence from Norway revealed 18 nucleotide differences. These included two synonymous and nine non-synonymous substitutions in protein-coding genes (dN/dS > 1): hence, the mitogenome of *L. pertusa* may be experiencing positive selection. To test for the presence of cryptic species, the mitochondrial control region and the nuclear ITS2 were sequenced for five individuals from each site: Italian and Norwegian populations turned out to share haplotypes of both markers, indicating that they belonged to the same species.

**Conclusions:**

*L. pertusa* corals collected 7,500 km apart shared identical nuclear ITS2 and near-identical mitogenomes, supporting the hypothesis of a recent connection between *Lophelia* reefs in the Mediterranean and in the Northern Atlantic. Multi-locus or population genomic approaches will be required to shed further light on the genetic connectivity between *L. pertusa* reefs across Europe; nevertheless, ITS2 and the mitochondrial control region may be useful markers for investigating the phylogeography and species boundaries of the keystone genus *Lophelia* across its worldwide area of distribution.

## Background

The mitochondrial genomes of cnidarians and sponges are characterized by low rates of evolution that make it often impossible to distinguish species using *cox1* sequences [1,2]. Moreover, the mitochondrial genomes of scleractinian corals exhibit a nearly perfect conservation of a standard gene order [3], in which non-coding regions and additional genes (such as duplicated tRNAs [4-6], putative homing endonucleases [7] and unknown ORFs [8]) are sometimes found inserted. The order of the basic complement of 13 protein-coding genes, 2 rRNAs and 2 tRNAs, all located on the same strand in scleractinian mitochondrial genomes, has been found to be identical in every species investigated so far except in the cold-water coral *Lophelia pertusa* (Linnaeus, 1758) [9,10].

*L. pertusa*, an azooxanthellate scleractinian coral, is an important deep-sea reef builder with an almost cosmopolitan distribution [11]. The reefs it builds host highly diverse and rich faunas [12-14], but these fragile ecosystems are being severely impacted by destructive fishing practice such as bottom trawling [15,16]; furthermore, CO_2_-induced ocean acidification may affect their future distribution [17] or even cause their disappearance [18]. Previous analyses of 16S rDNA mitochondrial sequences revealed a very high level of divergence (6.96%) between *L. pertusa* from the northeast Atlantic and off Brazil, suggesting that these two populations may represent cryptic species [19]. In the northern Atlantic, due to the lack of a mitochondrial marker variable enough for intraspecific studies [20], the population structure of *L. pertusa* has only been studied using internal transcribed spacer (ITS) sequences [21] and microsatellite markers [21-23]. This is unfortunate as mitochondrial markers present numerous advantages over nuclear ones: haploid markers are cheaper and easier to sequence (no heterozygosity issues), and have a smaller population size resulting in faster coalescence (thereby alleviating the problems posed by shared ancestral polymorphism). Hence we decided to sequence and compare the complete mitogenomes from two individuals originating in locations putatively isolated from each other (in the Barents Sea off Norway and in the Ionian Sea off Italy), in the hope of finding variable mitochondrial regions suitable for population genetic and phylogeographic analyses.

## Results and discussion

### The mitogenomes of *Lophelia pertusa* from the Mediterranean and Barents seas are nearly identical and may be experiencing positive Darwinian selection

**Figure 1:**
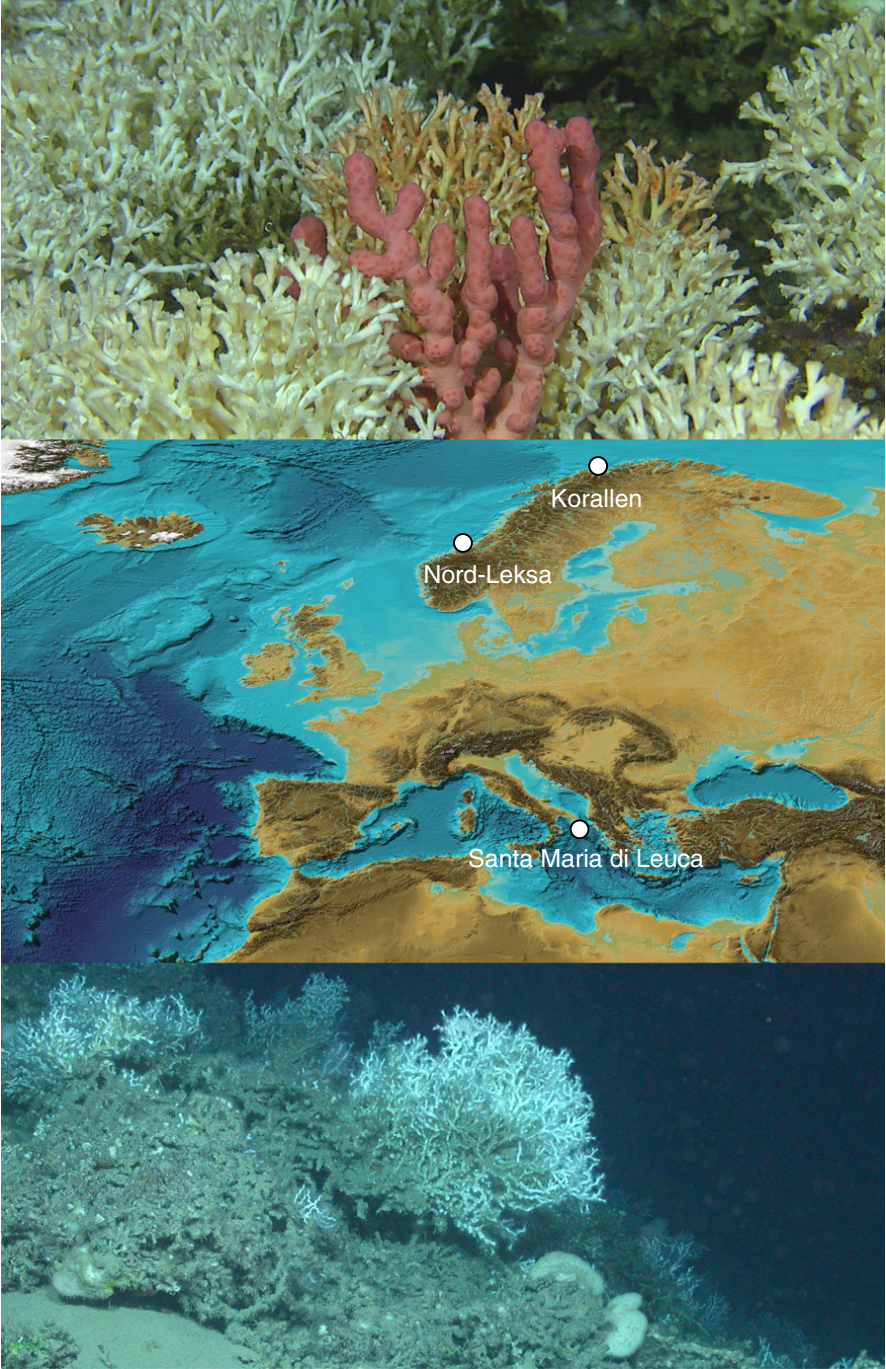
**Topographic map of Europe showing sampling locations.** The two populations sampled are Korallen off Norway and Santa Maria di Leuca off Italy. One field picture from each of the latter two sites was included in the figure (above, photograph from Korallen by Pål Buhl-Mortensen, Institute of Marine Research; below, photograph from Santa Maria di Leuca by Marum, University of Bremen). The published *L. pertusa* mitogenome [10] came from Nord-Leksa. The shaded relief comes from the ETOPO2v2 2-minute grid [24], which north of 64°N is based on the International Bathymetric Chart of the Arctic Ocean (IBCAO) [25].

In spite of the geographical distance between them (approximately 7500 km, Figure 1), the two *L. pertusa* individuals sequenced had nearly identical mitochondrial genomes that differed only by a nucleotide substitution at position 11,876. This single mutation (out of a total of 16,149 bp) is a transition; it is not silent but entails the substitution of a tyrosine (in individual #362 from Italy) with a histidine (in individual #302 from Norway) in the NAD6 protein. As such a mutation may be adaptive, we tested whether it was fixed in the two populations investigated by sequencing the corresponding DNA region in four additional individuals from each location (Table 1): the sequences of all eight individuals were identical to the one of individual #362, whereas the rare sequence variant of individual #302 was confirmed by resequencing.

**Table 1:** **Localization and depth of each *Lophelia pertusa *colony sampled**

**Sample code**	**Country**	**Markers sequenced**	**Coordinates**	**Depth (m)**
#295	Norway	Control region, *nad6*, ITS2	70°55.960 N, 22°12.333E	164
#302	Norway	Complete mitochondrial genome, ITS2	70°55.963 N, 22°12.339E	162
#306	Norway	Control region, *nad6*, ITS2	70°55.809 N, 22°11.453E	201
#308	Norway	Control region, *nad6*, ITS2	70°55.810 N, 22°11.452E	200
#327	Norway	Control region, *nad6*, ITS2	70°55.655 N, 22°11.378E	170
#275	Italy	Control region, *nad6*, ITS2	39°33.140 N, 18°13.170E	548
#276	Italy	Control region, *nad6*, ITS2	39°33.148 N, 18°13.163E	577
#277	Italy	Control region, *nad6*, ITS2	39°37.290 N, 18° 39.050E	671
#279	Italy	Control region, *nad6*, ITS2	39°38.070 N, 18°40.230E	679
#362	Italy	Complete mitochondrial genome, ITS2	39°33.890 N, 18°26.237E	610

Another mitochondrial genome of *L. pertusa* from Norway was recently published [10]. The individual sequenced in that study was sampled inside Trondheimfjord, a fjord known to harbor subpopulations of *L. pertusa* genetically differentiated from the subpopulations on the continental margin [21]. Indeed, there were 18 differences between our sequences and the published one, allowing us to assess the variability of the various stretches of the mitochondrial genome of *L. pertusa* (Table 2). The most variable region was a 730-bp long segment between the *nad5* and *cob* genes that had been previously singled out as the putative control region [10] as it was the longest non-coding stretch and contained three tandem repeats of a 51-bp pattern (consensus: CCTCCATCTATGCATGTGGAACCAGTTCCGGAGCTTTCTCAGGGTTTGATC). Hence, the *nad5*-*cob* intergenic region of the *L. pertusa* mitogenome matches all three criteria used to identify mitochondrial control regions: length, variability, and the presence of tandem repeats [8].

**Table 2:** **Variability of the mitochondrial genome of *Lophelia pertusa *(based on the comparison between our two sequences and **[10]**)**

**Region**	**Position**	**Number of AAs**	**Start codon**	**Stop codon**	**Transitions**	**Transversions**	**Indels**
*nad5(5*^*′*^*)*	1-714	238	ATG				
*nad1*	824-1771	315	ATG	TAA		N	
*atp6*	1824-2522	232	ATG	TAA			
*nad4*	2522-3967	481	ATG	TAG			
*rns*	3968-4991						
*cox3*	4992-5771	259	ATG	TAG			
*cox2*	5759-6484	241	GTG	TAA	N		
*nad4L*	6474-6773	99	ATG	TAA			
*nad3*	6775-7119	114	ATG	TAA			
*nad5(3*^*′*^*)*	7175-8296	373		TAA	N		
*control region*	8297-9026				2	1	1
*cob*	9027-10166	379	GTG	TAG		N, S, N	
*nad2*	10162-11478	438	ATT	TAA	N		
*nad6*	11480-12034	184	GTG	TAA	N, N		
*non-coding region*	12035-12415						1
*trnW*	12416-12485						
*atp8*	12489-12656	55	ATG	TAA			1
*cox1*	12646-14211	521	ATG	TAA	N, S		
*trnM*	14207-14277						
*rnl*	14278-16149				1		

Only the two largest non-coding regions are included in the table; synonymous mutations in coding regions are noted S, non-synonymous mutations are noted N.

Among the 11 nucleotide substitutions found in coding DNA regions, nine were non-synonymous and only two were synonymous (Table 2). We calculated dN/dS using ten different approaches (seven approximate and three based on maximum likelihood [26]). The values obtained ranged from 1.04 to 5.24 (Table 3), which is much higher than the value of 0.39 previously calculated in the shallow-water scleractinian coral *Pocillopora*[8]. Hence, the mitochondrial genome of *L. pertusa* appears to be experiencing positive (diversifying) Darwinian selection, whereas the mitochondrial genome of *Pocillopora* seems rather under negative (purifying) selection. However, this intriguing result will require further confirmation: the number of differences in coding sequences was so small that the support for positive selection was not statistically significant using Fisher’s exact test (other, more sensitive statistical procedures such as the Z test were not attempted as they require at least 10 synonymous and 10 non-synonymous mutations for their assumptions to be met [27]).

**Table 3:** **Computation of dN and dS between the mitochondrial protein-coding genes of #302 and **[10]**using 10 different approaches **[26]

**Method**	**Ka**	**Ks**	**Ka/Ks**	**P-value(Fisher)**
NG [28]	0.00100894	0.000742269	1.35927	0.965371
LWL [29]	0.0011027	0.000393124	2.80497	0.433222
MLWL [30]	0.00110429	0.000577782	1.91126	0.510732
LPB [31,32]	0.00122176	0.000233115	5.24101	0.105079
MLPB [30]	0.00112114	0.000466386	2.40389	0.343423
YN [33]	0.000998919	0.000768057	1.30058	0.961311
MYN [34]	0.000996698	0.000774126	1.28751	0.960344
GY-HKY [35,36]	0.000975458	0.000841603	1.15905	0.949187
MS [26]	0.000955594	0.000917088	1.04199	0.936033
MA [26]	0.000960752	0.0008924	1.07659	0.932721

The seven first methods in the table are approximate, and the three last ones are based on maximum-likelihood.

### European populations of *Lophelia pertusa* are comprised of two mitochondrial control region haplogroups but a single ITS2 field for recombination

Mitogenome divergence was higher between two individuals from Norway (our sequence and the published one) rather than between Norway and Italy (the two individuals we sequenced): as this raised the possibility that *L. pertusa* in Europe comprises two sympatric cryptic lineages, we sequenced the nuclear ITS2 and the mitochondrial control region, two markers of choice for species delimitation in corals [37-41], in a total of 10 individuals: five from Italy and five from Norway (including the two individuals whose complete mitochondrial genomes were sequenced in the present study).

**Figure 2:**
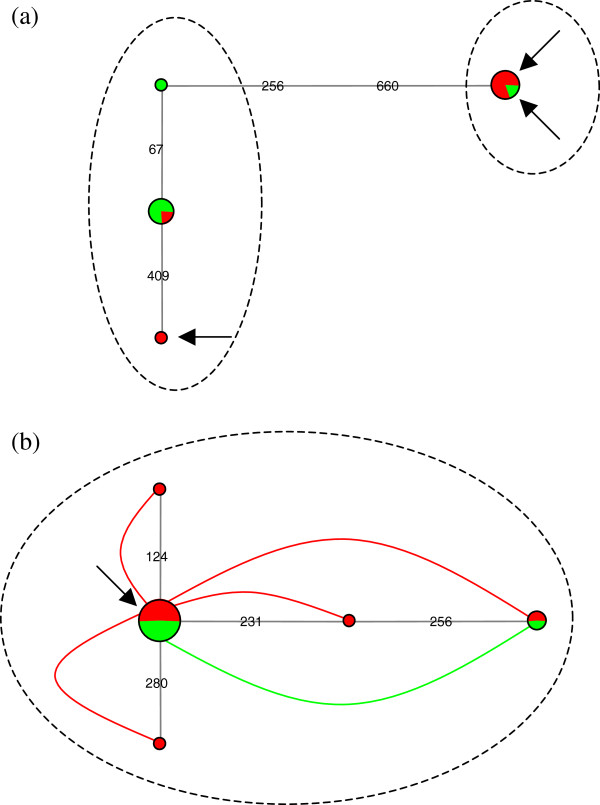
**Phylogeographic analysis of 10 *Lophelia pertusa *samples from Italy and Norway.** (**a**) Haplotype network (haplonet) of control region (CR) sequences. Green represents Italy and red Norway, whereas the numbers on the lines between haplotypes indicate the position of the corresponding mutations in the alignment (circle sizes are proportional to the number of individuals harboring each haplotype). Dashed ovals delineate two sympatric CR haplogroups (comprising respectively one and three haplotypes). The two arrows in the upper right corner point at the two individuals whose complete mitochondrial genomes were sequenced in the present study, whereas the bottom left arrow shows the CR haplotype in the published sequence of *L. pertusa*[10]. (**b**) Haplotype web (haploweb) of ITS2 sequences. The color code is the same as in (**a**), and curves connecting haplotypes represent heterozygous individuals harboring two different ITS2 types [39]. Circle sizes are proportional to the number of individuals harboring each type. The arrow points at the common ITS2 type shared by all individuals analyzed in the present study as well as by the one sequenced in [10,42], whereas the dashed oval delineates the resulting field for recombination [39,43].

There were three mitochondrial control region haplotypes among our 10 individuals (Figure 2a): the two major haplotypes, found in 5 and 4 individuals respectively, occurred both in Norway and in Italy, whereas a rarer haplotype was only found in one individual in Italy. The published sequence of *L. pertusa*[10] was one mutation away from one of the two most frequent haplotypes; overall, two haplogroups could be distinguished, comprising respectively one and three control region haplotypes.

There were five ITS2 sequence types among the 10 individuals analyzed: two of these types were shared by Italian and Norwegian populations, whereas the three others were only found in Norway. Several *L. pertusa* individuals contained two different ITS2 sequence types and one ITS2 type was shared by all individuals sequenced (Figure 2b), confirming that they belonged to a single field for recombination *sensu* Doyle [43] and were therefore conspecific following the criterion of mutual exclusivity [39].

Comparison of the ITS2 sequences obtained in the present study with those available online did not reveal any clear geographical pattern. The ITS2 type shared by all our samples (see arrow on Figure 2b) as well as by the coral colony whose mitochondrial genome had previously been sequenced [10,44] was also the most frequent type in [21] (found in 34 of the 77 samples of this study, from Norway to Spain). Our second most common type (on the right of Figure 2b), found in two heterozygotes from Italy and Norway, had been previously reported from France [21]. A rare ITS2 type that we found only in one heterozygous individual from Norway had been already observed in that country [21]. Only two rare ITS types from the present study, each found in Norway, had never been published until now.

### Shared mitochondrial and nuclear haplotypes support the hypothesis of a recent connection between Atlantic and Mediterranean *Lophelia pertusa* populations

The mitochondrial genomes of corals are known to be very stable compared with those of most other metazoans [1,2]. Identical sequences of a 630-bp fragment of the *cox1* gene was reported for populations of another azooxanthellate coral species, *Balanophyllia elegans*, sampled 3000 km apart [45], but it is the first time that such an extremely low level of variation across several thousand kilometers is reported using complete mitochondrial genome sequences. In contrast, the only other study that compared complete mitogenomes from conspecific corals reported between 3 and 18 intraspecific nucleotide differences in three *Montastraea* species analyzed at a single location [46].

The fact that *L. pertusa* populations located 7,500 km apart in different ocean basins share nuclear ITS2 and mitochondrial control region sequences contrasts with the results of previous population genetic studies using ITS and microsatellites that reported differentiation and even genetic discontinuities among northern Atlantic populations of this species [21,22]. A possible explanation could be a difference in time scale: at ecological time scale, differences in gene frequencies would indicate a low connectivity and/or predominantly asexual mode of reproduction of *L. pertusa* in the northern Atlantic, whereas at evolutionary time scale, the identical sequences found in individuals collected along the European shelf and in the Mediterranean Sea would point at a recent connection between these population. Indeed, the hypothesis of a Mediterranean origin for East Atlantic *L. pertusa* reefs was proposed by De Mol et al. [47,48] based on the correspondence between the depth distribution of coral mounds in the Porcupine Seabight and the depth of the Mediterranean outflow water: deep-sea reefs proliferated in the Mediterranean during the Younger Dryas (12,900 to 11,700 years BP) [49], a period during which the Mediterranean outflow was at its maximum [50] and could have carried coral larvae into the Atlantic. Our molecular results bring support to this hypothesis that will have to be further tested using multi-locus or population genomic approaches.

## Conclusions

*L. pertusa* individuals collected 7,500 km apart in the Barents and Mediterranean seas shared identical nuclear ITS2 and mitochondrial control region sequences, suggesting recent gene flow between *Lophelia* reefs in the Mediterranean and in the North Atlantic Ocean. Multi-locus or population genomic approaches will be required to shed further light on the genetic connectivity between *L. pertusa* reefs across Europe; nevertheless, ITS2 and the mitochondrial control region may be useful markers for investigating the phylogeography and species boundaries of the keystone genus *Lophelia* across its worldwide area of distribution.

## Methods

### Sample collection

*Lophelia* corals were sampled during research cruises at two distant localities (Table 1): Korallen (70°55^′^N & 22°12^′^E) is located northwest of the Norwegian island Sørøya in the Barents Sea, at the northern biogeographic limit for distribution of cold-water coral ecosystems [51], whereas the Santa Maria di Leuca coral province (39°33^′^N & 18°26^′^E) is found in the northern part of the Ionian Sea in the Mediterranean [52-54] (Figure 1). Both cruises (R/V Meteor Cruise 70/1 off Italy and R/V G.O. Sars Cruise No. 2006112 off Norway) were conducted in compliance with local legislations and with the Convention on Biological Diversity (CBD); samples were transported to Sweden with appropriate export and import permits following the Convention on International Trade in Endangered Species of Wild Fauna and Flora (CITES).

### Mitogenome sequencing and annotation

Frozen coral tissues were preserved in buffered guanidium thiocyanate solution [55,56] and their DNA purified on an ABI Prism 6100 Nucleic Acid PrepStation. PCR primers covering the entire genome of *L. pertusa* were defined using Primer3 [57] (Table 4). To prevent cross-contamination, the two mitochondrial genomes were not sequenced simultaneously but one after the other; moreover, we used filter tips and included negative controls in all our PCRs. PCR amplifications were performed in 25 μl reaction mixes containing 1x Red Taq buffer, 264 μM dNTP, 5% DMSO, 0.3 μM PCR primers, 0.3 units Red Taq (Sigma), and 10–50 ng DNA. PCR conditions comprised an initial denaturation step of 60 s at 94°C, followed by 45–55 cycles (30 s denaturation at 94°C, 30 s annealing, 1–3 min elongation at 72°C) and a final 5-min elongation step at 72°C. All PCR products were Sanger-sequenced using the same primers as for amplification (for long PCR products, internal sequencing primers were also defined using Primer3).

**Table 4:** **Primer pairs used for sequencing the mitogenome of *Lophelia pertusa***

**Forward primer**	**Reverse primer**	**Annealing T°**	**Fragment length**	**Reference**
5^′^-AAATCAAACGAGATTCCGAGAG-3^′^	5^′^-TCCATGGGGACTTCTCGTC-3^′^	53°C	1198 bp	this article
5^′^-TCGACTGTTTACCAAAAACATAGC-3^′^	5^′^-AAYAACCTTCCATTGCATCC-3^′^	53°C	1519 bp	this article
5^′^-TAGGAGTGGTTGGGAAATCG-3^′^	5^′^-CTTGGGGAAGCCAAATATGA-3^′^	53°C	2563 bp	this article
5^′^-GAACAACAGGGGCAACAGAT-3^′^	5^′^-ATGGTGTCCCTGAAAAGTCG-3^′^	53°C	2127 bp	this article
5^′^-GCAGACGCGGTGAAACTTA-3^′^	5^′^-TACCCCGGCTAAGACAACTG-3^′^	53°C	2551 bp	this article
5^′^-TTGTGGGGCAAATCATTCTT-3^′^	5^′^-AATGAGAAAGCCCACAAGCA-3^′^	53°C	1034 bp	this article
5^′^-CAACTCCGGTTTCTGCCTTA-3^′^	5^′^-TTTAAAAGAAAACTATGGAGGCCTAA-3^′^	53°C	3060 bp	this article
5^′^-TTATTGGGCCTGTGTTTGGT-3^′^	5^′^-CCCACATATGAAAAGGAGCAAC-3^′^	53°C	1604 bp	this article
5^′^-TGGGTGCTCTTTCTTCTGGT-3^′^	5^′^-AAATCCAATTGGTATATAATTTGTCA-3^′^	53°C	1237 bp	this article
5^′^-ATCCCTCCTTTTGCAGGATT-3^′^	5^′^-CCCCAGAAGCTGTTGTGTTT-3^′^	53°C	868 bp	this article
5^′^-GGCAATTGGTTCTGGGATAA-3^′^	5^′^-AAGCATACTAAAAGCCGTTCCA-3^′^	53°C	1254 bp	this article
5^′^-GGTCAACAAATCATAAAGATATTGG-3^′^	5^′^-TAAACTTCAGGGTGACCAAAAAATCA-3^′^	45°C	709 bp	[58]
5^′^-GCCGGTGCTATTACAATGCT-3^′^	5^′^-CAATCGATTCAAGCTCTTTTCA-3^′^	53°C	1892 bp	this article

As there was no published mitogenome of *L. pertusa* available at that time, the genes present in the two *L. pertusa* mitogenomes sequenced were identified using BLAST [59] and ORFfinder (available online at http://www.ncbi.nlm.nih.gov/gorf/orfig.cgi). One exception was the *atp8* gene that often cannot be annotated using BLAST since its aminoacid sequence is extremely variable; however, this gene always starts with the characteristic aminoacid sequence (M)PQ [60] and was therefore easily detected among translated ORF sequences. tRNA genes were localized using tRNA-scan [61], and tandem repeats were detected using the online program Tandem Repeats Finder [62]. Except for some details of gene boundaries, our annotation was consistent with the *L. pertusa* mitogenome that was published in the meantime [10].

### Haplonet and haploweb analyses

We sequenced the mitochondrial control region of four additional individuals from each site (Korallen and Santa Maria di Leuca), as well as the nuclear ITS2 of all ten individuals studied (see Table 5 for a list of the primers used). PCR conditions were as described in the previous section. The ITS2 chromatograms of five individuals comprised double peaks: obtaining the haplotypes of three of them was trivial as each of these individuals had only one double peak, and we used Clark’s method [42] to determine the haplotypes of the two remaining individuals that had two double peaks each. Although other authors have reported intra-individual mitogenome heterogeneity in *L. pertusa* using next-generation sequencing [44], we did not observe double peaks in the chromatograms obtained for any of the mitochondrial markers sequenced. The complete mitogenomes of individuals #302 and #362 were deposited in public databases [GenBank: KC875348-KC875349], as well as the CR and ITS sequences obtained in this study [GenBank: KC875351-KC875375]. Haplotype networks were built using the median-joining approach [63] implemented in Network 4.1 (available online at http://www.fluxus-engineering.com/); the ITS2 haplonet was turned into a haploweb by adding connections between alleles found co-occurring in heterozygous individuals [39].

**Table 5:** **Primer pairs used for phylogeographic analysis in *Lophelia pertusa***

**Marker**	**Forward primer**	**Reverse primer**	**Annealing T°**	**Fragment length**	**Reference**
ITS2 (nuclear)	5^′^-AGCCAGCTGCGATAAGTAGTG-3^′^	5^′^-GCTGCAATCCCAAACAACCC-3^′^	53°C	603 bp	[64]
CR (mitochondrial)	5^′^-AGGGGCCTTGTTCAATTTCT-3^′^	5^′^-AGGGAGAGGGCAAATTCACT-3^′^	53°C	941 bp	this article

## Competing interests

The authors declare that they have no competing interests.

## Authors’ contributions

MD initiated the study and obtained the samples, JFF sequenced and analyzed the markers and CA supervised the study. JFF drafted the manuscript that was revised by MD and CA. All authors approved the final version of the manuscript.
